# Supplementary material for: MetaRibo-Seq measures translation in microbiomes
Source: Nat Commun. 2020 Jun 29;11:3268. doi: 10.1038/s41467-020-17081-z (PMC7324362; doi:10.1038/s41467-020-17081-z)
Supplement: Supplementary file 10 — Supplementary Data 7 [file 41467_2020_17081_MOESM10_ESM.zip › File2/Confidence_High_Taxonomy/322658_out.krona.html]

Javascript must be enabled to view this page.

members
magnitude
magnitudeUnassigned
count
unassigned
taxon
rank

322658\_out

14

2
superkingdom
14

976
14
phylum

class
14
200643

171549
14
order

family
14
815

816
genus
14

817

SRS015890\_contig\_number\_1478SRS018313\_contig\_number\_9997SRS018984\_contig\_number\_19856SRS019267\_contig\_number\_6892SRS020869\_contig\_number\_32616SRS023346\_contig\_number\_3527SRS048981\_contig\_number\_3358SRS053573\_contig\_number\_3153SRS057717\_contig\_number\_3066SRS075021\_contig\_number\_1066SRS1055069\_contig\_number\_10614SRS143342\_contig\_number\_17950SRS147653\_contig\_number\_760
species
13

1
species

SRS024388\_contig\_number\_2583
671267
